# Estimating the impact of temperature and air pollution on cardiopulmonary and diabetic health during the TORONTO 2015 Pan Am/Parapan Am Games

**DOI:** 10.1186/1710-1492-10-S1-A62

**Published:** 2014-03-03

**Authors:** Laura Feldman, Jingqin Zhu, Jacqueline Simatovic, Teresa To

**Affiliations:** 1 Child Health Evaluative Sciences, The Hospital for Sick Children, Toronto, ON, Canada, M5G 1X8; 2 University of Toronto, Toronto, ON, Canada, M5S 1A1; 3 Institute for Clinical Evaluative Sciences, North York, ON, Canada, M5T 3M6

## Background

The TORONTO 2015 Pan Am/Parapan Am Games will attract thousands of visitors to Ontario, many of whom may suffer from chronic disease. It has been shown that those with asthma, asthma-related conditions, hypertension and diabetes are particularly sensitive to worsening air quality [1].

## Objective

To predict patterns of temperature, humidity and air quality, as well as health service use for cardiopulmonary conditions and diabetes in July 2015.

## Methods

Exposure data (temperature, humidity and air pollution) were obtained from Environment Canada for years 2003 to 2010. Using ArcGIS, the geospatial patterns of exposures were described for regions of Ontario hosting Pan Am events. A linear trend was used to forecast expected exposures for Pan Am regions in July 2015. Health outcomes (hospitalizations, emergency department visits and outpatient claims) for all-cause morbidity, asthma, asthma-related conditions, diabetes and hypertension were measured using data provided by the Institute for Clinical Evaluative Sciences. Associations between exposures and health outcomes were obtained from regression models. Health outcomes were predicted for July 2015 using scenarios of 5% and 10% higher exposure levels than forecasted.

## Results

**Figure 1:**
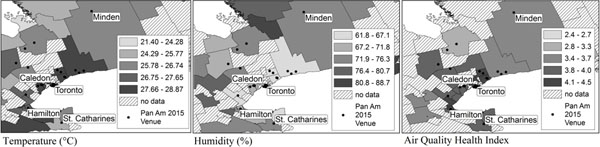
The geospatial distribution of temperature, humidity and Air Quality Health Index (AQHI)^a^ in Pan Am and Parapan Am regions of Ontario in July 2010. ^a^A composite measure of NO_2_, PM_2.5_ and O_3_ where 1-3=low health risk, 4-6=medium health risk, 7-10=high health risk.

Figure 1 shows the geospatial differences in temperature, humidity and air quality across Pan Am regions of Ontario in July 2010. Predicted daily rates of hospitalization and outpatient claims showed the largest increase under scenarios of increased exposure levels (Table 1). Given a 10% higher temperature than forecasted, predicted daily outpatient claims rates were 15% higher for all causes (Table 1), 20% higher for asthma and 20% higher for hypertension, compared to predicted rates using the forecasted temperature. Given a 10% higher Air Quality Health Index (AQHI) level than forecasted, predicted daily hospitalization rates were 6% higher for all causes (Table 1), 4% higher for asthma and 4% higher for asthma-related conditions, compared to predicted rates using the forecasted AQHI level.

**Table 1:** For Pan Am regions: observed and forecasted exposures, with predicted daily health service use rates for all-cause morbidity in July 2015 under scenarios of 5% and 10% higher exposure levels than forecasted

	Observed	Forecasted	Hospitalizations^c^			Emergency Department Visits^c^			Outpatient Claims^c^		
Exposures	(July ’03-’10)	(July ’15)	Forecasted	5%↑	10%↑	Forecasted	5%↑	10%↑	Forecasted	5%↑	10%↑
Temperature (°C) ^a^	25.66	25.74	3.36	3.62	3.88	10.95	10.99	11.02	253.46	272.30	291.15
Humidity (%)^b^	71.2	69.5	3.67	3.90	4.13	10.99	11.03	11.07	275.91	293.36	310.82
AQHI^a^	4.2	2.6	2.84	2.93	3.01	10.90	10.91	10.92	216.85	222.85	228.86

^a^Monthly average of daily maximums. ^b^Monthly average of daily averages. ^c^Per 10,000 general population.

## Conclusions

With thousands more people being exposed to Ontario’s weather and air pollution in July 2015, it is especially important to consider strategies to minimize the environmental impact of human activities. This will lessen the potential burden on individuals, especially those living with chronic disease.
